# A microfluidic technique to estimate antigen expression on particles

**DOI:** 10.1063/1.4989380

**Published:** 2017-10-09

**Authors:** Tanmay Ghonge, Anurup Ganguli, Enrique Valera, Mariam Saadah, Gregory L. Damhorst, Jacob Berger, Gelson Pagan Diaz, Umer Hassan, Monish Chheda, Zeeshan Haidry, Stan Liu, Carissa Hwu, Rashid Bashir

**Affiliations:** 1 Department of Bioengineering, University of Illinois at Urbana-Champaign, Urbana, Illinois 61801, USA; 2 Micro and Nanotechnology Lab, University of Illinois at Urbana-Champaign, Urbana, Illinois 61801, USA; 3 Biomedical Research Center, Carle Foundation Hospital, Urbana, Illinois 61801, USA; 4 Carle Illinois College of Medicine, Urbana, Illinois 61801, USA

## Abstract

Antigen expression is an important biomarker for cell analysis and disease diagnosis. Traditionally, antigen expression is measured using a flow cytometer which, due to its cost and labor intensive sample preparation, is unsuitable to be used at the point-of-care. Therefore, an automatic, miniaturized assay which can measure antigen expression in the patient could aid in making crucial clinical decisions rapidly. Such a device would also expand the use of such an assay in basic research in biology. In this paper, we present a microfluidic device that can be used to measure antigen expression on cells. We demonstrate our approach using biotin-neutravidin as the binding pair using experimental and computational approaches. We flow beads with varying biotin surface densities (*m_r_*) through a polydimethylsiloxane channel with cylindrical pillars functionalized with neutravidin. We analyze how shear stress and collision angle, the angle at which the beads collide with the pillars, affect the angular location of beads captured on the pillars. We also find that the fraction of captured beads as a function of distance (*γ*) in the channel is affected by *m_r_*. Using *γ*, we derive the probability of capture per collision with the pillar (*ε*). We show that *ε* is linearly related to *m_r_*, which is analogous to the expression level of proteins on cell surfaces. Although demonstrated with beads, this assay can next be expanded with cells, thus paving the way for a rapid antigen expression test.

## INTRODUCTION

Antigen expression on cells can be used as a marker for disease/infection diagnosis. For example, it has been reported that CD64 expression on neutrophils can be used as a biomarker for early diagnosis of bacterial infection.^1^ CD71 has been found to be upregulated on cancer cells of breast,^2^ colon,^3^ and lungs.^4^ Flow cytometry is the gold standard for measuring the expression level on cells. However, samples for flow cytometric analysis have to be manually stained with fluorophores, which makes the process time-consuming and labor-intensive.^5^ A rapid and automated test for antigen expression hastens appropriate clinical intervention and immensely improves patient-care.

Zhang and Pappas have reported a microfluidic device for estimating the surface expression ratio of cell types.^6^ In this device, CD71 expressing Ramos B lymphocytes and the CD71 expression ratio of lymphocytes in whole blood cells were immunologically captured in two separate channels. The number of captured cells was enumerated under a microscope. The ratio of cells captured in two channels was shown to be able to predict the CD71 expression ratio. However, it was unclear how the absolute value of surface expression could be determined using this assay. Vickers *et al.* reported a spiral microfluidic device for separating physically similar human umbilical vein endothelial cells and human microvascular endothelial cells based solely on the difference in CD31 expression.^7^ Our group has recently demonstrated a label-free, point-of-care device capable of estimating CD64 expression on neutrophils from a drop of blood.^8^ It was shown that the number of CD64+ neutrophils captured immunologically in a “capture chamber” varies linearly with the average expression of CD64 on neutrophils. White blood cells entering and exiting the channel were counted using a miniaturized coulter counter, paving the way for a hand-held, automated device.

In this paper, we demonstrate a simple microfluidic technique which can be used to optically measure the expression level of proteins on particles and cells. Central to our assay is an immuno-capture technique which has been shown to specifically capture cells of interest.^9^ For demonstrating the proof of concept, we used biotin-neutravidin as the binding pair. We flow biotinylated beads with varying surface densities (*m_r_*) in a microfluidic channel which has cylindrical obstacles functionalized with neutravidin. Adhesive interaction between the beads and the pillars captures the beads. First, we will observe how an interplay between the shear stress and the collision angle, the angle at which beads collide with the pillars, determines the angular location of a captured bead on a pillar. Thereafter, we show that the fraction of beads captured as a function of length (*γ*) can be used to obtain the probability of capture per collision (*ε*), which varies linearly with *m_r_* (*r*^2^ = 0.99). Although demonstrated with beads, this assay can just as easily be extended to estimate antigen expression on cells.

## RESULTS

**FIG. 1.**
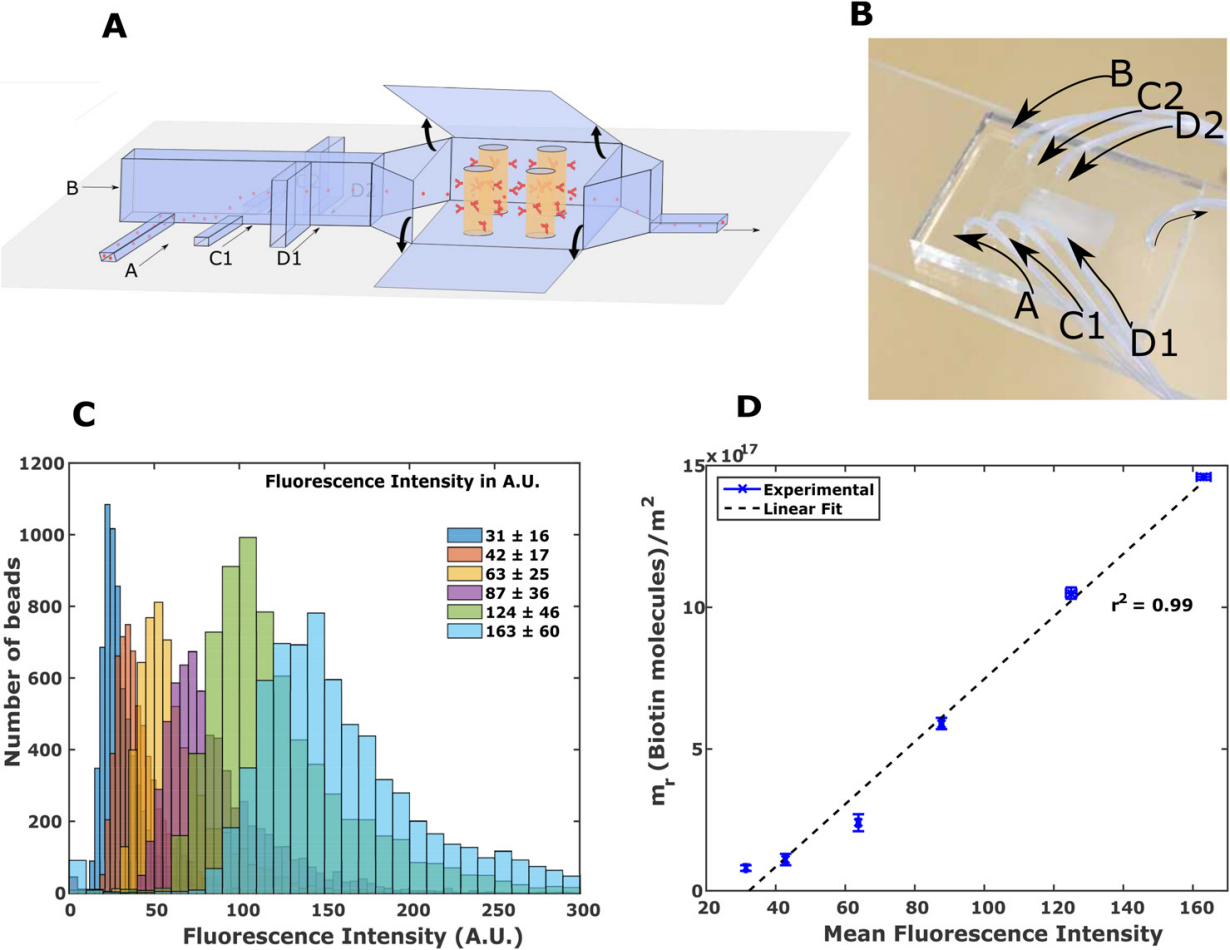
(a) Schematic of the microfluidic channel showing hydrodynamic focusing of beads in fluid stream A by buffers in fluid streams B, C1, C2 (not visible), D1, and D2 (partially visible). Buffer in streams B, C1, and C2 focuses the beads in the vertical direction. Buffer in streams D1 and D2 focuses the beads laterally. Beads are captured downstream on neutravidin coated cylindrical pillars. (b) Actual device showing all fluid streams. (c) Biotin surface density of six populations of beads is quantified by fluorescence assay using a flow cytometer. (d) The absolute surface density of biotin on the beads (*m_r_*) correlates linearly with the mean fluorescence intensity calculated (MFI) in (c). The error bars show the mean and standard deviation for 3 independent trials of the measurement of Mean Fluorescence Intensity and *m_r_*.

The schematic of the microfluidic channel used for the experiment is shown in Fig. 1(a), and an optical image of the fabricated devices is shown in Fig. 1(b). Beads are injected from port A at 1 *μ*l/min. We tested 6 populations of beads with varying surface densities of biotin (*m_r_*) [Fig. 1(c)]. To ensure that beads experience consistent shear stress, buffer from ports B, C1, C2, D1, and D2 hydrodynamically focuses the beads in the channel.^10^ At the end of the experiment, microfluidic channels are imaged under a microscope. Coordinates of the captured beads are extracted using a custom written macro in FIJI (see Methods for details).

### Precise channel design ensures predictable path of beads

**FIG. 2.**
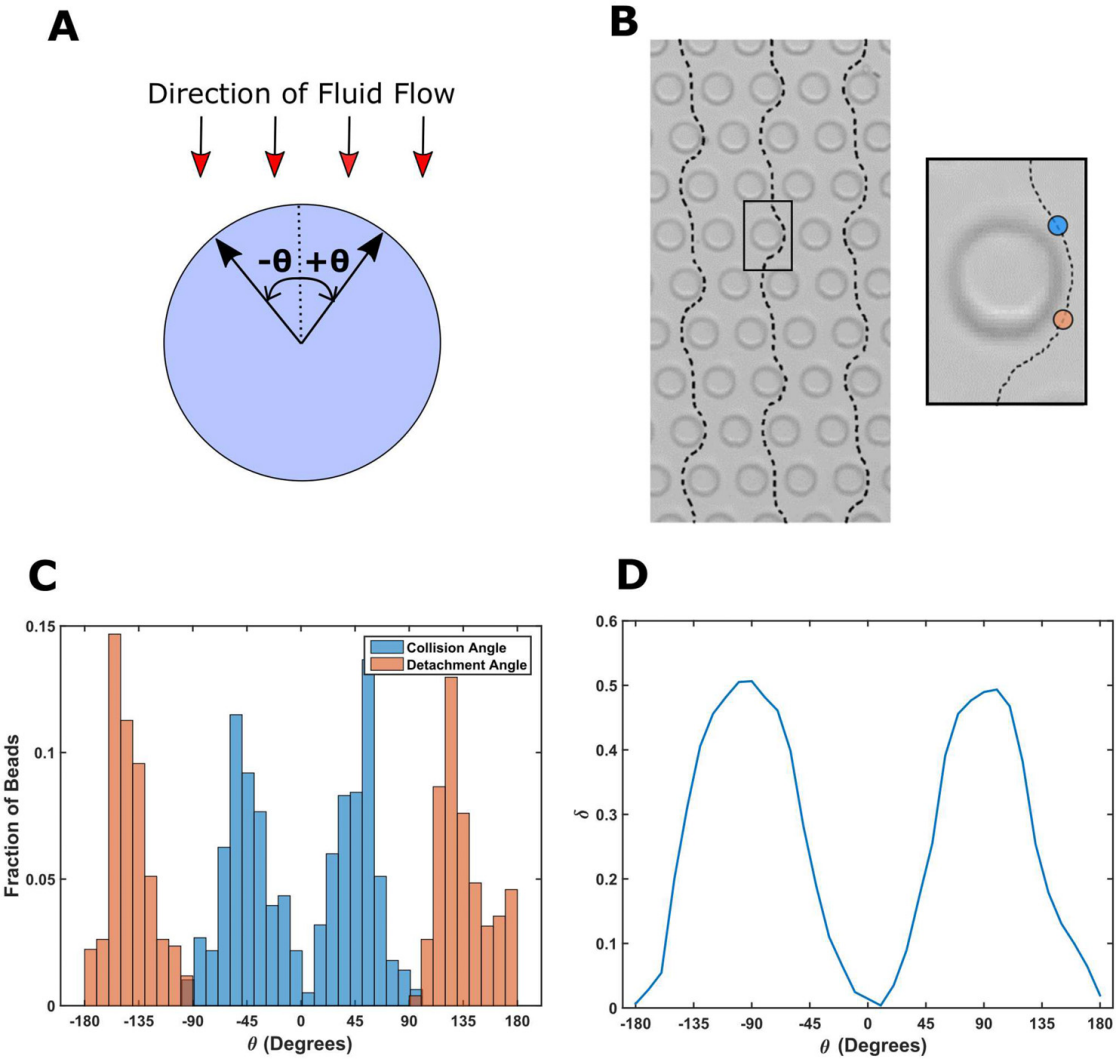
(a) The schematic shows a pillar with respect to the fluid flow. Black arrows show the sign convention used for describing angular locations. (b) shows a subset of particles tracked in the channel using a high speed camera. The inset shows the zoomed in view of a pillar. The angular location at which a bead makes the first contact with the pillar is called the “collision angle” (shown by the blue circle). The angular location at which a bead detaches from the pillar is called the “detachment angle” (shown by the orange circle). (c) shows the experimentally obtained histogram of the collision angle and the detachment angle. The histogram has been normalized such that the height of each bar represents the fraction of total beads by either colliding or detaching in a 10° bin width. (d) shows the experimentally calculated fraction of particles (*δ*) in contact with the pillar as a function of *θ*.

We designed the pillar geometry in such a way that the beads travel along a predictable path in the channel (see Methods for details). Figure 2(a) shows the sign convention followed to describe the angular location *θ* around a pillar. Figure 2(b) shows the trajectory of three beads tracked in a channel which was coated with bovine serum albumin (BSA) to prevent any non-specific interaction. Therefore, the trajectory is independent of *m_r_*. We observe that although the beads are displaced slightly on either side cyclically, they travel straight on average.^11^ The trajectory of the bead is cyclic, and it repeats after every three rows (supplementary material, Movie 1).

Figure 2(b) (right) shows the magnified view of the trajectory of one bead around a pillar. The blue circle represents the position at which the bead makes first contact with the pillar. We call the angle at which the bead collides with the pillar as the “collision angle.” Thereafter, the bead slides around the pillar, while still maintaining contact, until it reaches the position denoted by the orange circle. Here, the bead detaches from the pillar. The angle at which a bead detaches from the pillar is called the “detachment angle.” Figure 2(c) shows the experimentally obtained histogram of contact angle and the detachment angle. It has been normalized such that the height of each bar represents the fraction of total beads that either makes contact or breaks contact in a 10° bin-width.

At any given angle, the fraction of particles in contact is denoted by *δ*. For *θ* > 0°, the number of particles in contact (*N*) is calculated by subtracting the cumulative sum of the number of beads that have detached from the cumulative sum of the number of beads that have collided between 0° and *θ*. For *θ* < 0°, *N* is calculated by subtracting the cumulative sum of the number of beads that have detached from the cumulative sum of the number of beads that have collided between *θ* and 0°. *δ* is equal to *N* divided by the total number of beads. Figure 2(d) shows *δ* as a function of *θ.*

We assume that the trajectory of beads in a channel functionalized with neutravidin is not significantly different from that observed in the channel coated with BSA. This assumption is reasonable as, given the diameter of pillars, the gap between pillars, and the horizontal shift between two adjacent rows of pillars, the streamline followed by a bead is a function of size only.^12^ As a result, the collision angle histogram will remain the same regardless of the molecules present on the pillar. The histogram of the detachment angle is expected to slightly shift towards *θ* = ±180° as the beads would tend to remain in contact longer due to adhesive interactions. We ignore the effect of a small change on the detachment angle in our analysis.

### Interplay between collision angle and shear stress determines the angular capture location

**FIG. 3.**
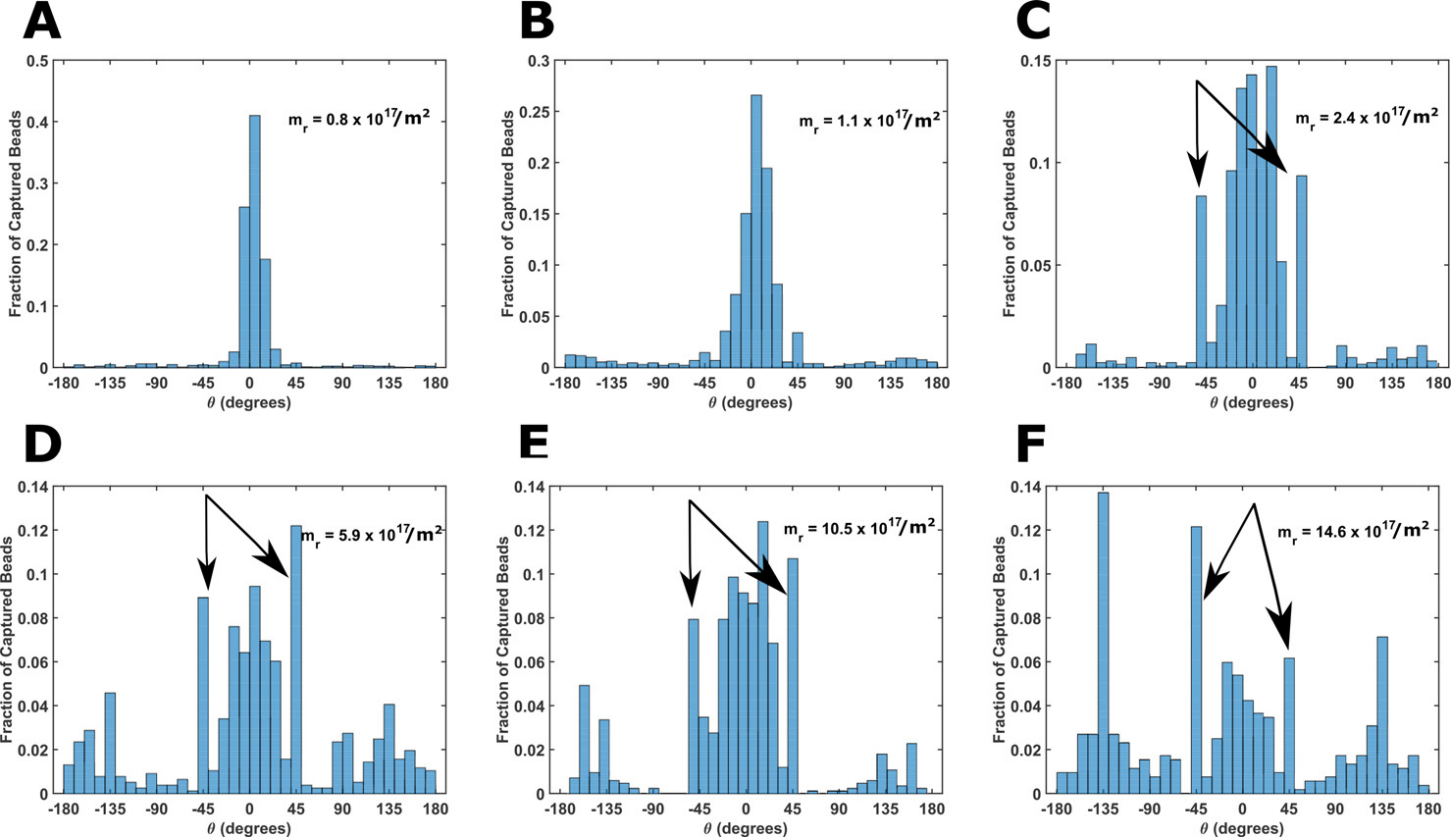
Interplay between shear stress and the collision angle determines the angle at which the beads are captured. The normalized histogram of the capture-angle for six populations [(a)–(f)] of biotinylated beads is shown. To eliminate steric effects of already captured beads, only those pillars were included in analysis which captured only one bead. Beads having a low surface density of biotin (*m_r_* = 0.8 × 10^17^) are captured exclusively around *θ* = 0°. This indicates that bead capture is dominated by shear stress effects. Local maxima of the capture-angle at ±45° [pointed at by arrows in (c), (d), (e), and (f)] become progressively more prominent with increasing *m_r_*. It coincides with the maxima of the collision-angle in (c). This indicates that as *m_r_* increases, the bead capture is dominated by collision frequency.

Figure 3 shows the histograms of the angles at which beads are captured on the pillars for different values of *m_r_*. The histogram has been normalized such that the height of the bar represents the fraction of total beads that were captured in 10° bin widths. We have considered only those pillars which captured just one bead. This eliminates the interference of an already captured bead in the angular location of the said bead. As *m_r_* increases, the histogram spreads out with the emergence of local maxima at *θ* = ±45°, pointed by arrows in Figs. 3(c)–3(f). In contrast, the maxima in Figs. 3(a) and 3(b) are at *θ* = 0°. This shift in the most preferred location of capture can be explained by the competing effects of shear stress and those of the collision angle.

For *m_r_* = 0.8 × 10^17^/m^2^ and 1.1 × 10^17^/m^2^, we hypothesize that the probability of capture outside the region |*θ*| < 10°, due to high shear stress (see Fig. S4, supplementary material), is negligibly low. Despite the fact that the chance of bead making a contact with a pillar in region |*θ*| < 10° is extremely low [Fig. 2(d)], more than 90% beads are captured here. Because the chance that the beads will touch the pillar in the region |*θ*| < 10° is low, beads have to travel very long distances in the channel before they collide with the pillar at an angle where shear stress is conducive for capture. Consequently, capture events are expected to be rare and spread far apart. This hypothesis will be verified in the Spatial Profile of Captured Beads section.

As *m_r_* increases, the probability increases that a bead will be captured at an angular location where it first makes contact with the pillar. A significant fraction of beads collide with the pillars at *θ* = ±45° [Fig. 2(c)]. This explains local maxima in Figs. 3(c)–3(f) at *θ* = ±45°. Maxima in Fig. 3(f) at *θ* = ±135° can be explained as follows. If a bead is not captured in the region |*θ|* < 45°, shear stress between 45° and 135° seems to be too high for the beads to be captured. However, the shear stress falls dramatically as the bead approaches *θ* = 135° (Fig. S4, supplementary material), and it is captured again.

Increasing the flow rate and decreasing *m_r_* reduce the probability of capture [Eq. (1)]. Hence, an increase in the flow rate or a decrease in *m_r_* is expected to have similar effects on the histogram of the capture-angle. Upon increasing the flow rate for beads with *m_r_* = 14.6 × 10^17^/m^2^, the relative prominence of beads captured at *θ* = ±45° decreases compared to the beads captured at *θ* = 0° (Figs. S5C and S5D, supplementary material). We would like to point out that the 10° bin-width does not have any physical significance. We choose the 10° bin width so that the trend of capture angle histograms with increasing *m_r_* is easily observed.

### Spatial profile of captured beads can determine the surface density of biotin

**FIG. 4.**
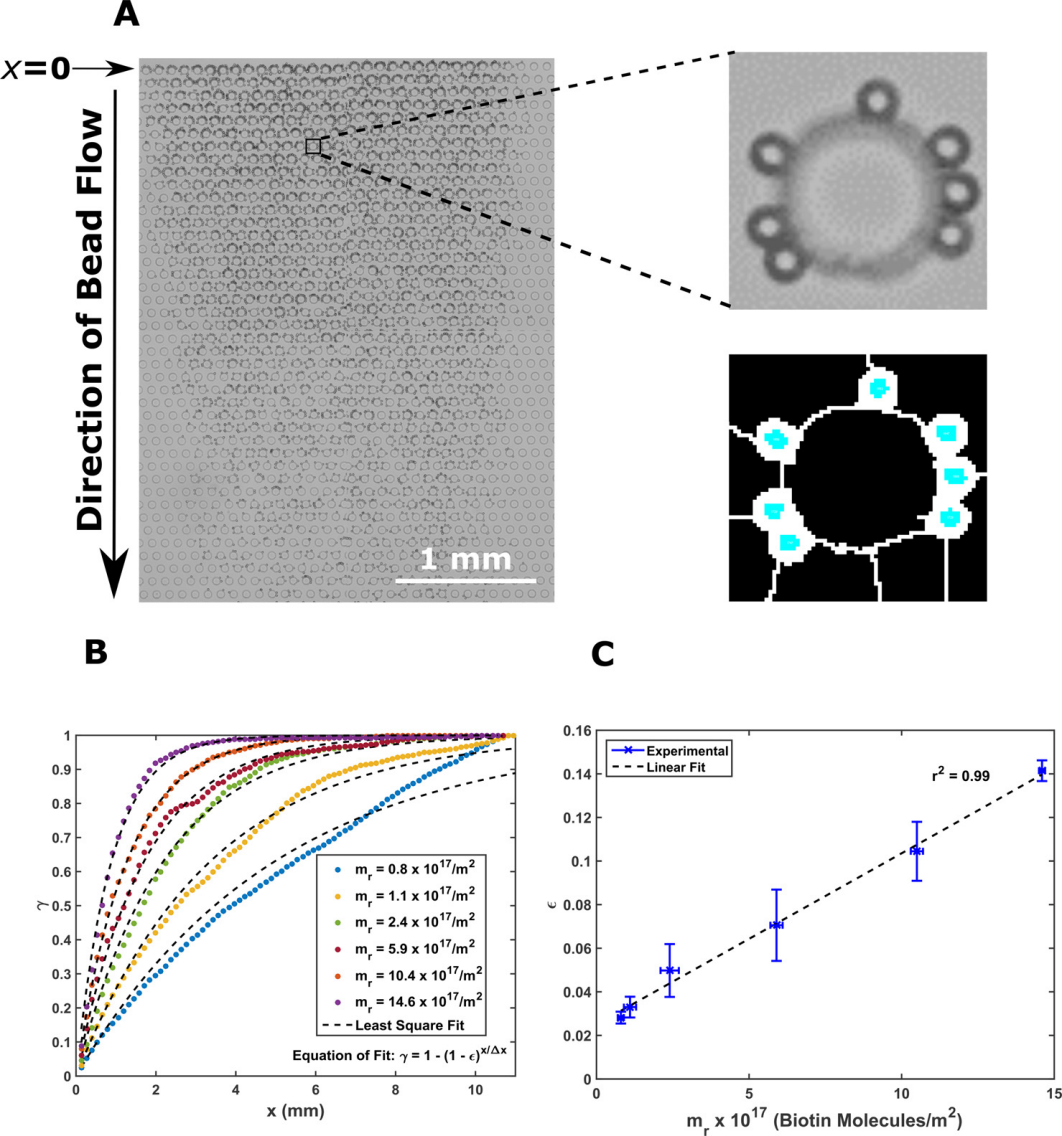
The spatial profile of captured beads predicts the surface density of biotin. (a) The image in the left shows the spatial distribution of captured beads in the channel for beads with *m_r_* = 14.6 × 10^17^/m^2^; the image in the right shows the zoomed view of one pillar and the corresponding image after bead identification with FIJI. (b) shows the fraction of captured beads, *γ*, as a function of the channel length, *x*, for six populations of beads tested in this study. We obtain *ε* from these curves by using least squares fitting. (c) shows the variation of the probability of capture per encounter, *ε*, as a function of biotin surface density (*m_r_*). *ε* has high linear correlation (*r*^2^ = 0.99) with *m_r_*. The error bars show the standard deviation for 3 independent trials.

Figure 4(a) shows the spatial profile of beads (*m_r_* = 14.6 × 10^17^/m^2^) captured in the channel. We observe that the number of beads captured per unit length gradually decreases along the direction of the flow of beads. A systematic study revealed that *m_r_* substantially affects the spatial profile.

In order to quantify this influence, we extract the probability of capture per interaction with a pillar, denoted by *ε*, from the experimentally acquired profile of captured beads. This is done by theoretically fitting the expected capture profile [Eq. (2)] to the experimental data observed fraction of beads captured in the channel (*γ*). A representative plot of *γ* for 6 bead populations tested in this study is shown in Fig. 4(b). As predicted by Eq. (2), *ε* increases linearly with *m_r_* [Fig. 4(c), *r*^2^ = 0.99]. Therefore, the surface density of biotin molecules can be accurately determined by analyzing the spatial profile of captured beads.

We hypothesized in the Interplay Between Collision Angle section that bead capture would be spread far apart for beads with lower *m_r_*. This hypothesis is verified in Fig. 4(b). Note that *γ* increases slowly for *m_r_* = 0.8 × 10^17^/m^2^ compared to *m_r_* = 14.6 × 10^17^/m^2^. According to Eq. (1), the probability of capture decreases with the increase in the flow rate. This is reflected in the decrease in the value of *ε* when the flow rate increases (Fig. S5B, supplementary material).

Although this technique for measuring surface density has been demonstrated with beads, we believe that it could be applied to the measurement of antigen expression on cells too, for example, measurement of CD64 on neutrophils. In order to take into account the variability in the size of neutrophils, the channel has to be designed in such a way that all the cells of interest travel in a straight line. For example, neutrophils' size varies between 10 and 15 *μ*m so that the gap between adjacent pillars should be set at 20–26 *μ*m so that they travel in a “zigzag” fashion.^11^ Since we have a low Reynolds number regime, we do not expect physical parameters other than the size, such as deformability or morphology, to affect the antigen expression measurement.

It is a well-known fact that antigen expression varies within a cell population. However, a limitation of this technique is that it measures only the mean surface density of molecules of cells. In a cell population with varying antigen expression, the profile of captured cells would resemble the profile of a cell population in which all the cells express the antigen equal to the mean antigen expression in the said cell population. Technical advancements in the technique might allow researchers to extract the range of antigen expression in a cell population. However, for diagnostic purposes at point-of-care, mean antigen expression on cells, like CD64 on neutrophils, still provides valuable insight into the disease state of a patient.

## CONCLUSIONS

We presented a microfluidic technique that can be used to measure the antigen expression or surface density of molecules on particles. We began with a geometry of pillars in which beads travel predictably and its trajectory repeats every three rows. Next, we observed that the angular distribution of captured beads around a pillar broadens as the surface density of biotin increases. This showed that the capture of beads is affected progressively more by the collision angle as compared to the shear stress. Next, we observed that the spatial profile of the captured beads is strongly affected by the surface density of biotin molecules. From this spatial profile, we obtained the probability of capture per collision, *ε*, which predicted the surface density of biotin accurately (*r^2^* = 0.99). Although this technique has been demonstrated with beads, it can just as easily be extended to cells. It is easy to implement as it requires commonly available laboratory instruments such as syringe pumps and a bright-field microscope or a cell phone camera. Hence, this technique can prove to be an inexpensive, rapid, and label-free way to estimate antigen expression on cells.

## METHODS

Ethics approval is not required for this study.

### Theoretical model for bead capture

The equation that governs the probability of particle capture, *P*, mediated by bond formation between receptors and ligands has the form^13^

$$P=m_{r}m_{l}A_{c}K_{a}(f/n),$$ (1)

where *m_r_* is the surface density of receptors (biotin), *m_l_* is the surface density of ligands (neutravidin), *A_c_* is the area of interaction between the biotinylated bead and the neutravidin coated pillar, and *K_a_(f/n)* is the binding affinity. It is equal to the ratio of forward and reverse rate coefficients for the formation and breakage of the nth bond, respectively. This equation has been used in the literature to estimate the probability of capture.^14^ It is assumed that the dislodging force, *f*, is equally shared by *n* bonds, where *n* is the smaller number of molecules available on two surfaces for bond formation, i.e., *n* = min (*A_c_m_r_, A_c_m_l_*). In this experiment, *m_l_* is less than *m_r_* (see supplementary material for the determination of *m_l_* and *m_r_*). So, *n* can be replaced by *A_c_m_l_* in the above equation. This equation predicts that the probability of adhesion increases linearly with receptor density (*m_r_*). As the flow rate increases, the dislodging force increases, while the probability of capture decreases.

If we assume that the downstream distance between consecutive pillars that a bead collides with is *Δx* and the chance of capture per encounter with a pillar is *ε*, then, a bead will encounter *x/Δx* pillars at a distance *x*, and the probability of capture *γ* can be expressed as

$$\gamma =1-{\left(1-\varepsilon \right)}^{x/\Delta x},$$ (2)

[probability of no capture on *n* encounters is (1 − *ε*)^*n*^, and subtracting it from 1 gives the probability of capture; here, *n = x/Δx*]. This model, however, does not take into account the bead-bead interaction. Through our experiments, we will acquire *γ* as a function of *x*. Thereafter, we fit the experimental data to this model to estimate *ε*. *ε* is expected to be linearly related to *m_r_* [Eq. (1)].

### Experimental scheme: Hydrodynamic focusing and circular geometry of pillars

Since the shear stress determines the probability of capture, it is necessary that all the beads experience identical stress. Moreover, for mathematical tractability, the shear stress experienced must be predictable along the length of the channel. The experiment was so designed that it fulfilled the above two conditions.

#### Hydrodynamic focusing

In a microchannel whose height is many times the size of the bead, the primary cause of the variability of the shear stress experienced by the beads on the pillars would be due to the variation in the vertical position of the bead in the channel. To ensure consistency, all the beads were brought to a uniform height in the channel by fluidic means.^10^ Figure 1(a) shows the schematic of the microchannel. Beads are injected from port A. Fluid injected from ports B, C1, and C2 (partially visible) focuses the beads in the vertical direction. The flow rate is adjusted such that all the beads move to the mid-point of the channel. The hydrodynamic focusing is simulated in COMSOL (supplementary material, Movie 2), where it is assumed that the height of channels A, C1, and C2 is very small as compared to that of channel B so that the 3D problem could be converted into a 2D problem. Now, all the beads are confined in one plane and do not experience the variation in shear stress due to the vertical location in the channel. Moreover, since all the beads are in one horizontal plane, they can be imaged more consistently. Fluid injected from ports D1 and D2 (partially visible) focuses the beads laterally. The flow rate of A was 1 *μ*l/min, B was 6 *μ*l/min, C1 and C2 was 3 *μ*l/min, and D1 and D2 was 6 *μ*l/min. The height of channels A, C1, and C2 is 15 *μ*m, while that of channels B, D1, and D2 is 60 *μ*m.

#### Pillar arrangement

A precisely designed cyclic arrangement of pillars ensures that the beads experience a predictably repeating dislodging force. Figure S1 shows the top view of the capture region. The gap, *g*, between two pillars is 14 *μ*m, and the pattern repeats every 3 rows. Motion in such a cyclic geometry depends only on the size of beads. 7 *μ*m sized beads (particle diameter/gap ratio of 0.5) travel in the “zigzag” mode^15^ following a cyclic procession of streamlines and make a contact with the pillars every 3 rows. Hence, *Δx* = 3 × (14 + 30) *μ*m =132 *μ*m. Because the motion of beads is cyclic, shear stress experienced by the beads is cyclic too (supplementary material, Movie 1).

### Biotinylated bead synthesis and surface density quantification

Beads with varying surface densities of biotin were prepared by linking Amine-PEG_2_-Biotin to Carboxylate Modified Latex beads via standard EDC-NHS [1-ethyl-3-(3-dimethylaminopropyl)carbodiimide hydrochloride - N-Hydroxysuccinimide] chemistry. The step-wise schematic for preparing the beads is shown in Fig. S2 (supplementary material). The full protocol can be found in the supplementary material. To confirm the presence of biotin on the beads, they were incubated with fluorescently labelled streptavidin (Thermofisher, Catalog No. SA1001) for 2 h at room temperature. After discarding the aspirate, the beads, suspended in 1× PBS (phosphate buffer saline), were analyzed using a flow cytometer. Figure 1(c) shows the fluorescence intensity of six populations of beads that were prepared.

The absolute concentration of biotin on the surface of the beads was also determined. The number of molecules consumed in the reaction was divided by the number of beads to obtain an estimate of average biotin molecules on each bead. The estimated average number of biotin molecules is divided by the surface area of a bead to obtain the surface density of biotin (*m_r_*). We made 6 populations of beads with *m_r_* = 0.8 × 10^17^/m^2^, 1.1 × 10^17^/m^2^, 2.4 × 10^17^/m^2^, 5.9 × 10^17^/m^2^, 10.5 × 10^17^/m^2^, and 14.6 × 10^17^/m^2^. The number of biotin molecules consumed in the reaction was determined by subtracting the number of unreacted molecules from the total number of molecules supplied at the start of the reaction. The number of unreacted molecules was estimated by using a Fluorescence Biotin Quantitation Kit (Thermofisher, Catalog # 46610). The standard curve for estimating the biotin concentration in the solution is provided in the supplementary material. The mean fluorescence intensity varies linearly with *m_r_* [Fig. 1(d)].

### Channel fabrication

The mold was made of SU8 by conventional lithography technique. The microfluidic channels were made of polydimethylsiloxane (PDMS) by using standard soft-lithography. The full protocol for channel fabrication has been described elsewhere.^9^

### Surface modification

#### Neutravidin functionalization

For bead capture experiments, channels were incubated with 100 μg/ml neutravidin (Thermofisher catalog No. 31000) and stored at 4 °C overnight to functionalize PDMS with neutravidin.^16^ Prior to the experiment, chips were incubated with 1% BSA (Thermofisher catalog No. 31000) for 1 h at room temperature to block the non-specific interaction. After each incubation step, chips were washed by flowing 100 *μ*l of 1× PBS.

#### BSA functionalization

To prevent non-specific binding, the channel was incubated with 1% BSA for 1 h at room temperature to block any interaction. To remove the unreacted molecules, chips were washed by flowing 100 *μ*l of 1× PBS.

### Data acquisition and analysis

The bead capture experiment was performed by using beads and flow rates described above in microfluidic channels functionalized with neutravidin. A total of 20 000 beads were injected in the channel for each experiment. All bead populations were assayed in triplicates. After the bead capture experiment, the still images of the channel were acquired with a 10× objective. Under this magnification, 24 images were required to cover the area where beads were captured. The images were then stitched together, and the physical location of each bead was determined using custom written macros in FIJI.^17^ The coordinates of beads so obtained were exported to an excel file for further analysis in MATLAB.^18^

To record the trajectories of beads around the pillars, the beads in the buffer were injected at flow rates described above in a channel blocked with BSA. The motion of beads was recorded at 2000 frames/s using a high speed camera (Vision Research Inc., Phantom v310). To obtain the coordinates of beads in each frame of the movie, a macro was written in FIJI. The coordinates of all the beads in all the frames were exported to an excel file for further analysis. We used the particle tracking code developed by John Crocker and Eric Weeks^19^ in MATLAB to track each bead in the channel.

### COMSOL simulations

We used COMSOL 5.1 to simulate the hydrodynamic focusing of particles and estimate the shear stress in the channel. All the simulations were performed in the Microfluidics Module using appropriate boundary conditions.

## SUPPLEMENTARY MATERIAL

See supplementary material for the protocol for fabricating beads with varying biotin surface densities, characterization of the surface density of biotin on beads and neutravidin on PDMS, and the effect of the flow rate on shear stress and capture probability.
